# Clinical observation of docetaxel or gemcitabine combined with cisplatin in the chemotherapy after surgery for stage II-III non-small cell lung cancer

**DOI:** 10.12669/pjms.315.7380

**Published:** 2015

**Authors:** Qiuqiang Chen, Xuexian Ji, Xiao Zhou, Qilin Shi, Huanming Yu, Hengqin Fu

**Affiliations:** 1 Qiuqiang Chen, Department of Thoracic Surgery, The Directly Affiliated Hospital of Huzhou Teachers College, Huzhou, Zhejiang 313000, China; 2 Xuexian Ji, Department of Pathology, People’s Hospital of Deqing County, Wukang, Zhejiang, 313200, China; 3 Xiao Zhou, Department of Respiratory Medicine, The Directly Affiliated Hospital of Huzhou Teachers College, Huzhou, Zhejiang 313000, China; 4 Qilin Shi, Department of Pathology, The Directly Affiliated Hospital of Huzhou Teachers College, Huzhou, Zhejiang 313000, China; 5 Huanming Yu, Department of Thoracic Surgery, The Directly Affiliated Hospital of Huzhou Teachers College, Huzhou, Zhejiang 313000, China; 6 Hengqin Fu, Department of Respiratory Medicine, The Directly Affiliated Hospital of Huzhou Teachers College, Huzhou, Zhejiang 313000, China

**Keywords:** NSCLC, Postoperative chemotherapy, Docetaxel, Gemcitabine, Cisplatin

## Abstract

**Objective:**

This study aimed to compare the efficacy and toxicity of docetaxel combined with cisplatin (DP) and gemcitabine combined with cisplatin (GP) in postoperative chemotherapy after surgery of non-small cell lung cancer (NSCLC).

**Methods:**

A total of 92 patients diagnosed with NSCLC after surgery were enrolled, and they were treated with DP (DP group) and GP (GP group). The efficacy and toxicity of the medications were then compared.

**Results:**

Approximately 92.4% (85 out of 92) of the patients received chemotherapy for more than three weeks. In DP and GP groups, the incidence rates of grade III-IV thrombocytopenia were 24.4% and 6.38%, respectively, whereas the incidence rates of alopecia were 88.9% and 25.5%, respectively. The difference between the two groups was statistically significant (*P* < 0.05). Disease-free survival rates in DP group in one and two years were 76.5% and 50.47%, respectively, whereas in GP group were 77.8% and 49.52%, respectively. No significant difference was observed between the two groups (*P* > 0.05).

**Conclusion:**

These results showed similar disease-free survival rates of DP and GP therapies in one and two years after surgery for NSCLC. However, DP group exhibited higher incidence of grade III-IV thrombocytopenia and alopecia than GP group. Therefore, we should select a specific treatment for each patient according to individual differences.

## INTRODUCTION

Lung cancer has become a life threatening disease with the highest incidence and mortality among cancer types in large and medium-sized cities of China; lung cancer is also recognised as one of the main causes of death in the world.^1^ Non-small cell lung cancer (NSCLC) accounts for 80% to 85% of lung cancer cases^2^ mostly diagnosed at stages II to III. A better efficacy in stage II to III NSCLC is observed in surgery-based chemotherapy or radiation therapy compared with chemotherapy or radiation therapy alone.^3^-^5^ Chemotherapy is an important part of combined therapy for NSCLC and can enhance the survival rate of patients subjected to surgery.^6^

Cisplatin-based adjuvant chemotherapy is recommended by the American Society of Clinical Oncology for adjuvant chemotherapy and adjuvant radiation therapy of stages II to IIIA NSCLC published on JCO in 2007.^7^ However, standard chemotherapy has not been applied in clinical research and the selection of appropriate chemotherapy remains a challenge. In current clinical studies, docetaxel or gemcitabine combined with cisplatin (DP or GP, respectively) is widely applied, thereby eliciting a definite effect for NSCLC, but only few retrospective comparisons of efficacy and toxicity between these two chemotherapies have been published.

Therefore, we collected data based on GP or DP treatment of NSCLC after surgery in two hospitals in 2004. We observed and analysed whether or not postoperative recurrence rates and toxic effects are possibly reduced. We also determined whether or not the treatment increases long-term survival to provide evidence for personalised treatment of NSCLC.

## METHODS

### Patient selection

From January 1, 2002 to December 31, 2007, patients with NSCLC who underwent surgery were selected according to the following standards: 1) age < 70 years; 2) PS 0 to 2; 3) expected survival longer than six months; 4) no chemotherapy, radiation therapy, or molecular targeted therapy before surgery; 5) operation method was pulmonary lobectomy or pneumonectomy plus lymphadenectomy; 6) pathological type was NSCLC; 7) postoperative pathological stage for TNM was II to III; 8) respiratory condition after surgery should be normal or only slight dyspnoea after minor activity; 9) patients were normal in blood routine test, hepatorenal function, and electrocardiogram (ECG); and 10) willing to undergo a follow-up study. This study was conducted in accordance with the declaration of Helsinki after approval from the Ethics Committee of the directly affiliated hospital of Huzhou Teachers College. Written informed consent was obtained from all participants.

### General data

Among 112 surgical patients with NSCLC, 5 patients who refused further treatment after surgery and 15 patients who did not finish three cycles of chemotherapy were excluded. The remaining 92 patients were randomised into DP and GP groups. A total of 45 patients aged 32 to 67 years (median age = 55 years) in DP group consisted of 38 males and 7 females, including 27 cases of adenocarcinoma, 17 cases of squamous carcinoma and one case of adenosquamous carcinoma. A total of 47 patients aged 31 to 67 years (median age = 56 years) in GP group consisted of 41 males and six females, including 29 cases of adenocarcinoma, 17 cases of squamous carcinoma and one case of adenosquamous carcinoma. The patients in the two groups did not show significant differences in clinical features (*P* > 0.05). All of the cases were not subjected to EGFR test.

### Therapeutic method

DP Group. Docetaxel 75 mg/m^2^ was added to physiological saline solution (250 ml). The resulting solution was intravenously instilled for one hour with ECG monitoring, d_1_. Cisplatin (75 mg/m^2^) was intravenously instilled from 1 d to 3 d, d_1–3_. To reduce body fluid retention at 1 d before the treatment, the patients were administered with 8 mg of oral dexamethasone twice per day for 3 d. GP Group. Gemcitabine (1250 mg/m^2^) was diluted in physiological saline solution (150 ml). The resulting solution was intravenously instilled for 0.5 h in d_1, 8_. Cisplatin (75 mg/m^2^) was intravenously instilled from 1 d to 3 d, d_1–3_. In these two therapies, one cycle covered 21 d and each patient received at least three cycles of chemotherapy to evaluate its efficacy and side effects. Tropisetron and other similar medications were routinely administered to stop vomiting during chemotherapy. Blood routine, hepatorenal function and electrolytes were reviewed twice per week during chemotherapy. The patients with <3.5 × 10^9^ leucocytes/L underwent granulocyte colony-stimulating factor treatment to increase the concentration of leucocytes. The patients who were subjected only to surgery did not receive any chemotherapy.

### Follow-up

Medical records were collected, including patient age, surgery date, and other pertinent data. These records were reviewed in clinic or followed up by telephone to record the time of recurrence and metastasis. Follow-up session was conducted twice per year until December 31, 2009 with a follow-up rate of 95.5% (five cases were lost). The median follow-up time was 679 days. The loss of follow-up, the survival cases until the last follow-up or the non-cancer-related death cases were processed as the censored value according to statistical analysis.

### Observation of indices

The patients underwent several examinations before the treatment and every two weeks after the treatment, including medical history, physical functioning, blood routine and hepatorenal function. Toxic response was graded according to CTCAEv3.0 criteria.^8^

The survival rates in one and two years were counted from the beginning of surgery until the death after the last follow-up or the loss of follow-up. Disease-free survival was monitored beginning from the last stage of primary lung cancer surgery to the local recurrence of lung cancer or distant metastasis. This factor should be confirmed by CT, MRI, ECT, PET-CT or biopsy.

### Statistical analysis

Statistical analysis was performed in SPSS10.0 software. X^2^ and Kaplan-Meier methods were used to calculate the survival rate. *P* < 0.05 was considered as statistically significant.

## RESULTS

### Toxic response

A total of 45 and 47 patients in DP and GP groups finished more than three cycles of chemotherapy, respectively. No patient died because of chemotherapy-related toxic effect. The efficacy evaluation was performed more than three weeks after the last chemotherapy. The most common toxic responses of the patients in the two groups included bone marrow suppression, gastrointestinal reaction and hepatic dysfunction. The incidences of leucocytopenia in DP and GP groups were 86.7% and 76.6%, respectively, in which the incidences of grade III-IV leucocytopenia were 20.0% and 17.0%, respectively, without a significant difference (*P* > 0.05).

A statistical difference (*P* < 0.05) was observed in the incidences of grade III-IV thrombocytopenia and alopecia between DP group (24.4% and 88.9%, respectively) and GP group (6.38% and 25.5%, respectively). The incidence of hepatic dysfunction in DP and GP groups were 26.7% and 19%, respectively, indicating no significant difference (*P* > 0.05). Allergic response did not occur in two groups. No statistical differences in haematological toxicity, diarrhoea and rash (*P* > 0.05; Table-I).

**Table-I:** Comparison of toxic response between two groups.

Toxic response	DP		GP	
	I~II	III~IV	I~II	III~IV
Leukocytopenia	30	9	28	8
Thrombocytopenia	22	11	17	3
Decrease of haemoglobin	9	1	11	0
Hepatic dysfunction	12	0	9	0
Nausea and vomiting	22	0	20	0
Alopecia	32	8	10	2
Allergy	0	0	0	0

### Survival rate

Kaplan-Meier method was used to calculate the survival rate. Follow-up period ranged from 10 to 42 months with a median of 22 months. The median OS of all of the patients was 883 days (95% CI 687 to 1027 days) and the overall survival rates in one, two and three years were 85.5%, 64.17% and 49.61%, respectively. The median DFS of all patients was 619 days (95% CI 411 to 827 days) and the disease-free survival rates in one and two years were 75.6% and 45.0%, respectively.

### Survival rate comparison

The median survival in DP and GP groups were 889 and 880 days, respectively. The survival rates in one, two and three years were 93.97%, 75.39% and 50.47% in DP group and 94.76%, 74.58% and 49.47% in GP group, respectively, indicating no significant difference (*P* > 0.05). No statistical differences (*P* > 0.05) were also observed in disease-free survival rates in one and two years between DP group (76.5% and 50.47%, respectively) and GP group (77.8% and 49.52%, respectively).

## DISCUSSIONS

Surgery has been considered as the main treatment of stage II-III NSCLC, in which tumours are completely resected by expanding the range of patients with stage II-III NSCLC. At present, new adjuvant chemotherapy has been applied to patients diagnosed with stage III NSCLC to obtain definite efficacy. Cisplatin has been considered as one of the most important drugs to treat advanced NSCLC with a single-agent efficiency of 16% to 20%.^9^ In recent years, the advent of several new chemotherapy drugs, including paclitaxel and gemcitabine combined with cisplatin for chemotherapy, have made great progress in NSCLC treatment.

Docetaxel, classified as a broad-spectrum botanical antitumour and anticancer drug extracted and synthesised from needle-like leaves of yew trees, can form stable non-functional microbundles by strengthening microtubule polymerisation and inhibiting microtubule depolymerisation to inhibit mitosis of tumour cell.^10^ Studies have shown that the combined treatment of docetaxel significantly increases the disease control rate of NSCLC and extends the progression-free survival of patients compared with docetaxel alone, but toxic responses are evidently enhanced.^11^ In the case of combined docetaxel and platinum, the patients can develop dose-restricted bone marrow suppression as manifested by leucocytopenia.^12^

Gemcitabine is a new type of synthetic pyrimidine nucleoside analogues with broad-spectrum antitumour activity.^13^ Combined treatment of cisplatin and gemcitabine can increase their cytotoxic effects, thereby improving the efficiency of NSCLC treatment.^14^

Liu et al.^15^ compared the efficacy of three chemotherapies, including navelbine, docetaxel or gemcitabine combined with cisplatin in the treatment of advanced NSCLC by prospective, open and randomised study, but results did not show any significant difference. Some studies revealed that the efficiency of NVB, TAX or GEM combined with DDP in the treatment of advanced NSCLC ranges between 30.0% and 44.4% with a median survival ranging from 11 to 17 months; survival rate ranges from 35.0% to 61.0%, but these results did not have statistical difference.^16^-^18^ Although GEM combined with platinum has not been used in adjuvant chemotherapy for stage III NSCLC yet, data from the treatment of advanced NSCLC and several clinical trials involving stage II NSCLC with GEM have indicated that the combined treatment of GEM with platinum is more efficacious than NVB therapy in terms of safety and efficacy of postoperative chemotherapy.^19^-^21^ This treatment might become a standard scheme of postoperative chemotherapy for NSCLC.

In our research, a total of 92 patients with stage II-III NSCLC received postoperative chemotherapy of DP or GP. Disease-free survival rates in one and two years were 76.5% and 50.47% in DP group and 77.8% and 49.52% in GP group, respectively, but no significant difference was observed between the two groups (*P* > 0.05). Toxic responses, including bone marrow suppression and gastrointestinal reaction were observed in the two groups. Gastrointestinal reaction was associated with cisplatin. The incidence of grade III-IV thrombocytopenia in DP group was 24.4% and this incidence was higher than that of GP group (6.38%; *P* < 0.05). The incidence of alopecia in DP group was also higher than that of GP group.

The results showed a similar disease-free survival rate and overall survival rate for DP and GP chemotherapy for the treatment of stage II-III NSCLC. However, DP group exhibited higher incidence of grade III-IV thrombocytopenia and alopecia than GP group. Therefore, we should select a specific treatment for each patient according to individual differences.
